# Obstetric Anesthesia Practice in the Tertiary Care Center: A 7-Year Retrospective Study and the Impact of the COVID-19 Pandemic on Obstetric Anesthesia Practice

**DOI:** 10.3390/jcm11113183

**Published:** 2022-06-02

**Authors:** Paweł Krawczyk, Remigiusz Jaśkiewicz, Hubert Huras, Magdalena Kołak

**Affiliations:** 1 Department of Anesthesiology and Intensive Care Medicine, Jagiellonian University Medical College, Kopernika 17, 31-501 Cracow, Poland; 2 Department of Anesthesiology and Intensive Care Medicine, University Hospital, Jakubowskiego 2, 30-688 Cracow, Poland; rjaskiewicz@su.krakow.pl; 3 Department of Obstetrics and Gynecology, Jagiellonian University Medical College, Kopernika 23, 31-501 Cracow, Poland; hubert.huras@uj.edu.pl (H.H.); magdalena.kolak@uj.edu.pl (M.K.)

**Keywords:** obstetric anesthesia, cesarean section, vaginal delivery, spinal anesthesia, epidural anesthesia, general anesthesia

## Abstract

There are many benefits of neuraxial anesthesia (NA) in the obstetric population. We performed a retrospective analysis of anesthesia provided to obstetric patients in the tertiary care center between 1 January 2014 and 31 December 2020 and the influence of the COVID-19 pandemic on anesthetic practice. A total of 15,930 anesthesia procedures were performed. A total of 2182 (17.52%) cesarean sections (CS) required general anesthesia (GA), including 383 (3.07%) of emergency conversion from NA. NA for CS consisted of 9971 (80.04%) spinal anesthesia (SA) and 304 (2.44%) epidural anesthesia (EPI). We found a decrease in the GA rate for CS in 2020 (11.87% vs. 14.81%; *p* < 0.001). The conversion rate from NA to GA for CS was 2.39% for SA and 31.38% for EPI. The conversion rate from labor EPI to SA for CS increased in 2020 (3.10% vs. 1.24%; *p* < 0.001), as well as the SA rate for other obstetric procedures (61.32%; *p* < 0.001). We report 2670 NA for vaginal delivery, representing 31.13% of all vaginal deliveries. NA constituted the vast majority of obstetric anesthesia. However, we report a relatively high incidence of GA. There was a decrease in GA use in the obstetric population during the pandemic. Further reduction in GA use is possible, including an avoidable conversion from NA to GA.

## 1. Introduction

The growing number of obstetric procedures, including cesarean sections [1], requires anesthesia and recognition of its unique approach to the safety and expectations of the obstetric population. General anesthesia, although sometimes inevitable, carries a risk for both mothers and newborns. The main complications resulting from general anesthesia use in the obstetric population include difficult airway, aspiration of gastric content, awareness, and impaired neonatal adaptation after cesarean delivery [2,3,4]. The Society for Obstetric Anesthesia and Perinatology recently proposed recommendations for Centers of Excellence for Anesthesia Care of Obstetric Patients to reduce the overall general anesthesia rate to ideally ≤ 5%. The recommendations also included establishing a review of the quality assurance for the anesthesia care of obstetric patients [5]. The recommendations of the Royal College of Anaesthetists indicate an even lower rate of 1% for elective cesareans and less than 5% for the emergent cesarean section [6]. Both institutions recommend monitoring the general anesthesia rates for cesarean section in obstetric units as part of quality improvement programs. The risk of exposure of medical personnel to airborne particles during the COVID-19 pandemic gave an additional argument for reducing the use of general anesthesia if possible [7]. The provision of labor analgesia differs between countries, ranging from 5–74% of all vaginal deliveries in developed countries and has a multifactorial origin [8]. The aim of this study was to present seven years of obstetric anesthesia practice in the tertiary care center and its change during the COVID-19 pandemic.

## 2. Materials and Methods

After receiving approval from the Ethics Committee of Jagiellonian University, Cracow, Poland (approval number: 1072.6120.40.2021) and institutional approval, we conducted a retrospective study that included all obstetric procedures that required anesthesia in the obstetric tertiary care center (University Hospital, Cracow, Poland) from 1 January 2014 to 31 December 2020. Obstetric procedures were divided into cesarean section, vaginal delivery, and other obstetric procedures (manual removal of retained placenta, inspection, and suturing of the perineum). Anesthesia procedures were classified as general anesthesia, spinal anesthesia, epidural anesthesia, combined spinal–epidural, and intravenous anesthesia.

A Chi-square test was used to compare qualitative variables. Results with a *p*-value < 0.05 were considered significant. The analyses were performed using the R software (ver. 4.1.0.; R Foundation for Statistical Computing, Vienna, Austria) [9].

## 3. Results

**Figure 1:**
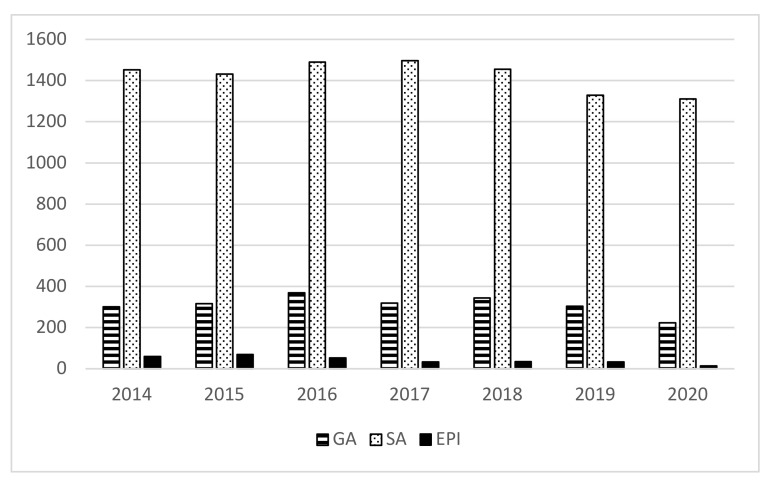
Anesthesia for cesarean sections. GA—general anesthesia; SA—spinal anesthesia; EPI—epidural anesthesia.

A total of 15,930 anesthesia procedures were performed (Table 1). There were 12,457 cesarean sections. A total of 2182 (17.52%) required general anesthesia, including 383 (3.07%) cesarean sections with the emergency conversion from neuraxial to general anesthesia. A total of 10,275 cesarean sections were performed with neuraxial anesthesia, including 9971 (80.04%) spinal anesthesia and 304 (2.44%) epidural anesthesia (Figure 1).

We found a decrease in the cesarean section general anesthesia rate in 2020 compared with previous years (11.87% vs. 14.81%; *p* < 0.001). The average conversion rate from neuraxial to general anesthesia for cesarean section was 2.39% (*n* = 244) for spinal anesthesia and 31.38% (*n* = 139) for epidural anesthesia. The conversion from labor epidural to spinal anesthesia for cesarean section was increased in 2020 compared with previous years (3.10% vs. 1.24%; *p* < 0.001). We reported 2670 neuraxial anesthesia for vaginal delivery, constituting 31.13% of all vaginal deliveries, including 2233 epidural anesthesia, representing 26.04% of all vaginal deliveries. Other obstetric procedures for manual removal of retained placenta, inspection, and suturing of the perineum required 803 anesthesia procedures, including 135 (16.81%) general anesthesia and 178 (22.17%) intravenous anesthesia. We recorded a decrease in the general anesthesia rate for other obstetric procedures in 2020, with more frequent use of spinal anesthesia 61.32% (*p* < 0.001). Detailed results are presented in Table 2.

## 4. Discussion

The presented data shows that neuraxial anesthesia constitutes the vast majority of obstetric anesthesia. However, there is a relatively high percentage of general anesthesia use in the obstetric population. The use of general anesthesia for cesarean section is the result of different factors that limit the application of neuraxial anesthesia, such as urgency (category 1) of cesarean delivery, low platelets, confirmed coagulopathy, unknown coagulation status in patients at risk of coagulopathy, or lack of a sufficient time interval from the last dose of low-molecule weight heparin [10], hypovolemia, and hemodynamic or respiratory failure. Many of these are common in tertiary care center obstetric patients compared with primary care centers.

Our study reports a relatively high incidence of general anesthesia of 14.81% for cesarean section; this may be due to different reasons. There are 2500–3000 deliveries per year in the tertiary care center and up to 10% of them are preterm deliveries. Larger centers with over 1500 annual births have higher general anesthesia rates ranging up to 20% in emergency cesarean sections [11]. Another risk factor for a higher general anesthesia rate is preterm delivery, which increases by 13% the adjusted odds of general anesthesia for cesarean section for every 1-week decrease in gestational age at delivery [12]. During the COVID-19 pandemic, we found a significant reduction to 11.87% of the general anesthesia rate for cesarean section. Data from the United Kingdom report a national cesarean section general anesthesia rate of 8.75%, with a possible regional further decline during the peak of the pandemic from 7.7% to 3.7% in selected centers. The authors recognized anesthetic decision making, the presence of an anesthetic consultant in the delivery suite, and the recommendations from anesthetic guidelines as the key factors that influenced this decline [13].

A systematic approach involving multidisciplinary team collaboration and implementation of a growing body of obstetric anesthesia evidence in clinical practice may limit the number of cesarean sections under general anesthesia, effectively reducing their incidence [14].

Obstetric anesthetists play an important role in reducing the number of avoidable general anesthetics for cesarean section. When planning anesthesia for obstetric patients, it should be proactive not reactive. Predicting and preventing urgency should characterize good cooperation between all healthcare providers taking care of parturient women, including the anesthesia team, obstetricians, midwives, and neonatologists. It requires training, drills, and simulation of emergencies [15].

It is essential to plan and prepare, to actively involve a multidisciplinary team when dealing with an obstetric emergency, to set priorities, and to simultaneously design the safe and optimal management for both mother and newborn, avoiding unnecessary exposure to general anesthesia. Implementing the recent consensus statement of the Society for Obstetric Anesthesia and Perinatology addressing the risk of complications of neuraxial anesthesia in obstetric patients with thrombocytopenia [16] will facilitate a safe approach to providing regional anesthesia in obstetric patients with thrombocytopenia; especially useful when balancing the risk of difficult airway and aspiration [17].

The 2009 statement of the Polish Society of Anesthesia and Intensive Care Medicine on guidelines for obstetric analgesia services indicates a safe platelet level for neuraxial procedures ≥100,000 × 106/L [18]; it is still valid, which may result in considerable caution in undertaking neuraxial anesthesia in Poland for obstetric patients with thrombocytopenia.

The Royal College of Anaesthetists proposed a standard of the target for best practice regarding the emergency conversion rate from regional to general anesthesia up to 1% for elective; <15% for category I and <5% for the category I–III cesarean sections [6].

We report a conversion rate from spinal anesthesia to general anesthesia for a cesarean section of 2.39% regardless of the urgency of the cesarean section, which appears to be acceptable. However, 31.38% of the failure of the labor analgesia top-up for cesarean delivery, regardless of the urgency of the cesarean section, needs improvement compared with the maximum failure rate of 25% of the category I cesarean section epidural top-up presented in the prospective audit [19].

We found an almost three-fold increase in conversion from a labor epidural to spinal anesthesia for the cesarean section in 2020, which may be a preventive measure to avoid general anesthesia in case of risk of labor epidural top-up failure for the cesarean section also recognized in other centers [6].

Strategies and interventions that allow reducing the conversion rate include early detection of a failing labor epidural, testing density and extent of the neuraxial block before surgery, modification in topping up epidural, location of top-up administration (delivery room vs. operating theater), use of local anesthetics and adjuvants with short onset of action, and anticipating and planning deliveries with potential complications.

We found several patients with intravenous anesthesia used for other obstetric procedures without securing the airway with a tracheal tube. Their number decreased in the last two years of the study with a significant reduction during the pandemic. In addition to the risk of exposure to airborne particles for medical personnel during the pandemic, it also carries the risk of gastric content aspiration for obstetric patients. However, this issue is a subject of ongoing debate in light of recent evidence regarding gastric emptying during delivery and postpartum and the use of gastric ultrasound in the obstetric population. This point-of-care ultrasound examination allows to estimate the potential risk of aspiration resulting from gastric content volume assessment; however, further development is needed in the obstetric patient population to provide evidence allowing to identify women at low risk of aspiration relevant for airway management with supraglottic airways [20].

We reported 2670 neuraxial anesthesia for vaginal delivery, representing 31.13% of all vaginal deliveries. The rate of epidural anesthesia for vaginal delivery was 26.04% of all vaginal deliveries. There was a relatively high percentage of spinal anesthesia for vaginal delivery commonly used in advanced labor. The combined spinal–epidural technique was not common in labor anesthesia practice.

The provision of labor analgesia is different in developed countries, ranging from only about 5% in Japan, followed by 5–38% in the Netherlands, 33% in the UK, 65% in the USA, and 74% in Belgium [8]. It has a multifactorial origin resulting from women’s own preferences and maternal expectations, area of residence, socioeconomic status, maternal birthplace, heterogeneity of access to labor analgesia, number of vaginal births, cultural differences, knowledge of safety in pain relief during childbirth and influence of epidural labor analgesia on the course of labor, maternal comorbidity, as well as local customs and medical practice [21,22,23,24]. In the Norwegian register study, the provision of epidural analgesia for labor ranged from 9.3 to 53.8% according to the maternal birthplace [24].

Since 2015, the labor anesthesia service has been reimbursed in Poland by the National Healthcare System. However, there was only a temporary increase in labor analgesia procedures in that year that was not observed in the following study period. It appears to be different from the financial reason for the low number of labor epidural analgesia. The relatively low rate of labor epidural in our institution may result from various reasons such as women’s own preferences and maternal expectations, the number of vaginal births, the knowledge of safety in pain relief during childbirth, and the influence of epidural labor analgesia on the course of labor. Unfortunately, many patients and healthcare providers still believe in the negative influence of epidural analgesia on labor progress.

In the authors’ institution, all women in labor are offered volatile analgesia with nitrous oxide at an early stage of labor and some also receive non-opioid intravenous analgesia. For epidural labor analgesia, bolus of low concentration high volume of either 0.1–0.2% ropivacaine or 0.075–0.125% bupivacaine mixed with fentanyl is the common regimen. In case of need for a top-up, the labor epidural for cesarean delivery 2% lignocaine mixed with bicarbonate, fentanyl, and epinephrine is used. In case of contraindications for epidural analgesia and the patient request for additional analgesia regimens, the PCA remifentanil intravenously may be offered; however, off-label administration of remifentanil for labor is not a common practice.

The study is not without limitations. It was a single-center retrospective observational study. The retrospective nature of the study generates an inferior level of evidence compared with prospective studies. We had to rely on the accuracy of an already collected institutional database of healthcare records. Limitations include the risk of selection bias, measurement errors, and the approach to the anesthesia care of obstetric patients provided by individual healthcare providers. The study results should be generalized with caution, and only association and not causation can be inferred from the study results. Admissions to tertiary care centers are related to pregnancy complications that can influence delivery mode. We do not provide information on maternal baseline characteristics, the urgency of reported cesarean sections based on the category (elective/emergency/urgent), and the newborn outcome. Anesthesia complications and the overall outcome are also beyond the scope of the manuscript.

## 5. Conclusions

Neuraxial anesthesia constituted the vast majority of obstetric anesthesia. However, we reported a relatively high incidence of general anesthesia use for obstetric procedures. During the COVID-19 pandemic, there was a decrease in general anesthesia used in the obstetric population. Further reduction in general anesthesia use is possible, including an avoidable conversion from regional to general anesthesia.

## Figures and Tables

**Table 1:** Obstetric procedures that required anesthesia.

Procedure	2014	2015	2016	2017	2018	2019	2020
Vaginal delivery *	332 (29.72%)	478 (36.23%)	401 (32.73%)	353 (26.20%)	357 (27.56%)	389 (32.99%)	360 (32.90%)
Cesarean sections	1816	1819	1914	1852	1837	1669	1550
Other obstetric procedures	101	129	124	116	123	104	106

* Value in brackets shows the percentage of the total number of vaginal deliveries.

**Table 2:** Type of anesthesia for obstetric procedures.

	Cesarean Section						
Anesthesia	Year						
	2014 (*n* = 1816)	2015 (*n* = 1819)	2016 (*n* = 1914)	2017 (*n* = 1852)	2018 (*n* = 1837)	2019 (*n* = 1669)	2020 * (*n* = 1550)
GA	246 (13.55%)	242 (13.30%)	293 (15.31%)	280 (15.11%)	296 (16.10%)	258 (15.46%)	184 (11.87%)
SA	1447 (79.68%)	1408 (77.41%)	1459 (76.23%)	1467 (79.21%)	1435 (78.22%)	1308 (78.37%)	1263 (81.48%)
EPI	61 (3.36%)	70 (3.85%)	54 (2.82%)	34 (1.84%)	36 (1.96%)	34 (2.04%)	15 (0.97%)
SA >> GA	39 (2.15%)	51 (2.80%)	41 (2.11%)	22 (1.19%)	31 (1.68%)	27 (1.62%)	33 (2.13%)
EPI >> GA	17 (0.94%)	24 (1.32%)	35 (1.83%)	18 (0.97%)	18 (0.98%)	20 (1.20%)	7 (0.45%)
EPI >> SA	6 (0.33%)	24 (1.32%)	32 (1.67%)	31 (1.67%)	21 (1.14%)	22 (1.32%)	48 (3.10%)
	**Other Obstetric Procedures** **(Manual Removal of Retained Placenta, Inspection, and Suturing of the Perineum)**						
**Anesthesia**	**Year**						
	**2014** **(*n* = 101)**	**2015** **(*n* = 129)**	**2016** **(*n* = 124)**	**2017** **(*n* = 116)**	**2018** **(*n* = 123)**	**2019** **(*n* = 104)**	2020 * **(*n* = 106)**
GA	13 (12.87%)	22 (17.05%)	23 (18.55%)	25 (21.55%)	26 (21.14%)	17 (16.35%)	9 (8.49%)
IVA	28 (27.72%)	31 (24.03%)	31 (25.00%)	30 (25.86%)	35 (28.46%)	15 (14.42%)	8 (7.55%)
SA	48 (47.52%)	51 (39.53%)	45 (36.29%)	53 (45.69%)	45 (36.58%)	46 (44.23%)	65 (61.32%)
EPI	11 (10.89%)	23 (17.83%)	23 (18.55%)	6 (5.17%)	15 (11.20%)	21 (20.19%)	19 (17.93%)
SA >> GA	0 (0.00%)	1 (0.78%)	0 (0.00%)	0 (0.00%)	1 (0.81%)	1 (0.96%)	0 (0.00%)
EPI >> GA	1 (0.99%)	0 (0.00%)	1 (0.81%)	0 (0.00%)	1 (0.81%)	2 (1.92%)	1 (0.94%)
EPI >> SA	0 (0.00%)	1 (0.78%)	1 (0.81%)	2 (1.72%)	0 (0.00%)	2 (1.92%)	4 (3.77%)
	**Vaginal Delivery**						
**Anesthesia**	**Year**						
	**2014** **(*n* = 332)**	**2015** **(*n* = 478)**	**2016** **(*n* = 401)**	**2017** **(*n* = 353)**	**2018** **(*n* = 357)**	**2019** **(*n* = 389)**	**2020** **(*n* = 360)**
SA	63 (18.98%)	64 (13.39%)	56 (13.96%)	73 (20.68%)	44 (12.33%)	56 (14.40%)	63 (17.50%)
EPI	269 (81.02%)	412 (86.19%)	342 (85.29%)	275 (77.90%)	311 (87.11%)	331 (85.08%)	293 (81.40%)
CSE	0 (0.00%)	2 (0.42%)	3 (0.75%)	5 (1.42%)	2 (0.56%)	2 (0.52%)	4 (1.10%)

GA—general anesthesia; IVA—intravenous anesthesia; SA—spinal anesthesia; EPI—epidural anesthesia; CSE—combined spinal–epidural; SA >> GA—conversion from spinal to general anesthesia; EPI >> GA—conversion from epidural to general anesthesia; EPI >> SA—conversion from epidural to spinal anesthesia. * Differences between 2020 and previous years are statistically significant (*p* < 0.001).

## Data Availability

Not applicable.
